# Metabolic profile in first episode drug naïve patients with psychosis and its relation to cognitive functions and social cognition: a case control study

**DOI:** 10.1038/s41598-023-31829-9

**Published:** 2023-04-03

**Authors:** Samir El Sayed, Sarah Gomaa, Alaa Alhazmi, Ibrahem ElKalla, Dalia Khalil

**Affiliations:** 1 Department of Psychiatry, Faculty of Medicine, Mansoura University, Mansoura, Egypt; 2 Mansoura University Students’ Hospital, Mansoura University, Mansoura, Egypt; 3 Department of Psychiatry, Hayat National Hospital, Riyadh, Kingdom of Saudi Arabia; 4 Faculty of Medicine, Mansoura University, Mansoura, Egypt; 5 Faculty of Medicine, Zagazig University, Zagazig, Egypt; 6 Present Address: Riyadh City, Riyadh Kingdom of Saudi Arabia

**Keywords:** Neuroscience, Health care, Medical research

## Abstract

1st episode drug naïve patients with psychosis might be at higher risk for cardiometabolic disturbances which could affect the different cognitive, and executive functions and domains of social cognition. This study aimed to study the metabolic parameters in 1st episode drug naïve patients with psychosis, to evaluate the relation of these cardiometabolic domains to the cognitive, executive functions, and social cognition. Socio-demographic characteristics of 150 first episode drug naïve patients with psychosis and 120 matched healthy control groups were collected. The current study also assessed the cardiometabolic profile and cognitive functions in both groups. Social cognition was examined by Edinburgh Social Cognition Test. The study revealed a statistically significant difference in parameters of metabolic profile among the studied groups (p < 0.001*), the scores of cognitive and executive tests were statistically significantly different (p < 0.001*). In addition, the patient's group has lowered scores of domains of social cognition (p < 0.001*). Also, the mean affective theory of mind was negatively correlated with the conflict cost of the Flanker test (r = -.185* p value = .023). The total cholesterol level (r = − 0.241**, p value = .003) and level of triglycerides (r = − 0.241**, p value = 0.003) were negatively correlated with the interpersonal domain of social cognition, the total cholesterol level is positively correlated to the total score of social cognition (r = 0.202*, p value = 0.013). Patients with 1st episode drug naïve psychosis showed disturbed cardiometabolic parameters which have deleterious effects on cognitive functions and social cognition.

## Introduction

First-episode psychosis (FEP) is a severe disabling psychiatric disorder characterized by psychotic and negative manifestations with cognitive dysfunctions. Its pathophysiology is not fully explained, metabolic abnormalities (MAs) are common comorbidities in these patients leading to a higher burden of medical diseases and complications. The average life expectancy of men and women with psychosis is, respectively, 15 and 12 years shorter than for those without psychosis^1^, in which metabolic and cardiovascular complications are considered crucial concerns^2^. FEP patients are at increased risk for metabolic and cardiovascular diseases, including hypertension, type II diabetes mellitus, and coronary artery disease^3,4^, in which different MAs have been identified as high-risk factors. Moreover, MAs have been linked to poorer functional consequences^5^, disturbing quality of life^6,7^, and non-compliance with psychotropic medications^8^ in these patients. Previous studies showed that first-episode patients with psychosis on therapeutic or minimal psychotropic intake usually have a higher prevalence of MAs, including lipid profile dysregulation, reduced uric acid levels, and elevated homocysteine and prolactin levels in comparison to the healthy population^9,10^.

Studies showed an elevated risk of cardiovascular mortality, 2.5 times higher in individuals with First Episode Psychosis (FEP) compared to age-matched healthy control^11,12^.

These patients are also four to five times more likely to be smokers^13^ with a higher risk of developing obesity up to twice those of the general population^14^.

Also, patients on antipsychotic medications, particularly obesogenic agents such as Olanzapine and Clozapine, can increase weight and elevate blood glucose and fasting triglycerides levels^15,16^. However, Zhang et al.^17^ showed glucose disturbances in first-episode drug-naïve patients with psychosis, concluding that psychotropic is not only the contributing factor leading to these adverse consequences but also may combine with underlying psychopathology factors, particularly, disturbed glucose homeostasis^18^, glucose intolerance, and insulin resistance^19^.

Disturbance of cognitive functions has been commonly described in FEP including attention span^20,21^, psychomotor speed^22,23^ mental flexibility^24,25^, working memory^26,27^, and other executive functions^28,29^. Cognitive dysfunctions are reported during the prodromal stage of psychosis. The previous studies confirmed impaired performance in drug naïve patients with psychosis compared to healthy individuals including verbal memory, speed of processing, and working memory which were comparable to those reported in antipsychotic-treated patients^27^. The researchers concluded that cognitive disturbances might determine the patient's consequences and also combine with prominent functional impairments in different domains of life including social, occupational, and daily living activities^30,31^.

On the contrary, normal cognitive function is highly associated with better clinical outcomes in FEP. A systematic review found that cognitive disturbances are associated with negative and disorganized domains of psychotic features rather than positive and depressive ones^32^. Other studies showed that patients who have higher cognitive performance might have less severe negative manifestations of a psychotic disorder^33,34^. In the same vein, there was a relation between neurocognitive performance and cognitive reserve (CR) which affected the outcomes of FEP^35–38^.

The evidence that 1st episode psychosis was associated with an activated immune system is growing, so neuroinflammation has been considered a potential underlying cause of cognitive dysfunctions in those patients^39^.

The researchers concluded that biological causes including altered neural reward systems and shared genetic loci between diabetes and duration of 1st episode psychosis were evident^40^.

Also, poor health domains including poor diet, sedentary lifestyle, smoking, and substance use were common in antipsychotic naïve patients^41^. All the biological causes are associated with a higher rate of overweight/obesity^42^, higher insulin resistance, and increased levels of total and low-density lipoprotein cholesterol, and triglycerides^43^.

Social cognition has referred to a dimension including perception, interpretation, and processing of information for adaptive social contexts^44^. It is composed of four subdomains: emotional processing which has been defined as the ability to perceive and experience inner emotions. Theory of mind (ToM) has made the individual attribute and represent the mental states of other people. Social perception has included decoding and interpreting social contexts in others, and attributional bias has referred to the generalized errors made when people evaluate or try to find reasons for their own and other's behaviors^45^.

Patients with first-episode psychosis (FEP) have deficits in social cognition^46^ and metacognition^47,48^ which include how to think about their own and others' mental experiences^49,50^.

## Patients and methods

### Settings

This study was conducted in Hayat National Hospital, Psychiatry Department, Riyadh, Kingdom of Saudi Arabia. The study was conducted from September 2020 to December 2021.

### Study design

This current methodological design is a cross-sectional observational case–control study aimed to investigate the metabolic parameters in 1st episode drug naïve psychotic episode, also to study if those metabolic domains have associations to the cognitive, executive, and social cognitive functions, also to assess the risks of developing the cognitive and social cognition impairments in these patients.

### Study population

This study was composed of 2 groups:

- The case group: a convenience sample of 150 patients of 1st episode drug naïve psychotic disorders including schizophrenia, schizophreniform, and brief psychotic disorder according to DSM 5^51^. Two expert consultants’ psychiatrists confirmed the diagnosis of patients with psychosis.
- A healthy control group composed of 120 participants of the employees of the Hospital matched with the case group regarding age, educational level, and sex.

### Inclusion and exclusion criteria

All participants of both sexes, with an age range from 18 to 60 years, who can read and write to complete the scales were included in the study.

Those that have a history of major psychiatric disorders, or other serious general medical conditions or are under the effect of psychotropic medications were excluded from the study.

## Methods

*Sociodemographic and clinical characteristics* this included age, sex, residence educational level, marital status, smoking, duration since onset of psychosis, and family history of psychosis.

*Positive and negative symptoms scale*^52^: The PANSS is a 30-item clinician-administered rating scale. It adapted 12 items from the Psychopathology Rating Schedule (PRS) and 18 items from the Brief Psychiatric Rating Scale (BPRS). It includes 3 subscales: Positive Scale, Negative Scale, and General Psychopathology Scale. Each item is rated with 1 to 7 points ranging from absent to extreme. The range for the Positive and Negative Scales is 7–49, and the range for the General Psychopathology Scale is 16–112.

*Cognitive and executive assessment* The Psychology Experiment Building Language^53^: The Psychology Experiment Building Language (PEBL) is a free, open-source software system that allows researchers and clinicians to design, run, and share behavioral tests. This battery included up to 70 cognitive tests, this study used the following tests:

A-Eriksen’s Flanker Test^54^: which was published in 1974, is a set of tests to assess selective attention, reaction time, and inhibitory function. In this task, the target is positioned in the center and is flanked by nontarget stimuli. The individual is requested to press the left or right arrow key according to the target’s direction.

B-GO /and No-go Test: Go/No-Go testing^55^: is often used as a component of a behavioral neurological examination to assess inhibitory control, reaction time, language processing, and selective attention. A classic example is to hold out two fingers, the index and middle fingers (palm down), and say to the examinee: “When I do this (showing two fingers in the form of a “V”), you do this (showing only the index finger), and when I do this (showing the index finger only), you do this (showing two fingers in the form of a “V”).

C-Simon Test^23^: this is a task in which participants are required to respond to a non-spatial feature (e.g. color, shape) of a lateralized stimulus by pressing one of two response buttons that are lateralized in the same spatial arrangement. Although stimulus position is irrelevant to task performance, the reaction time (RT) is longer when the response side is spatially incompatible with the stimulus position (incompatible stimulus–response (S-R) mappings) than in trials that require an ipsilateral response relative to stimulus position (compatible S-R mappings).

D-Tower of London Test (TOL)^56^: In the computerized version of the ToL the goal state is presented in the upper field of the screen. To match the goal configuration, the participants operate on the start state in the lower half of the screen by using a computer mouse to move the balls. Participants were told to transform the start state into the goal state in a predetermined minimum number of moves while following three rules:^1^ only one ball may be moved at a time, (2) a ball in the lower row cannot be moved when another ball is lying above, and (3) three balls may be placed on the tallest peg, two balls on the middle peg, and one ball on the shortest peg. TOL is used to assess strategy, problem-solving, color processing, hand–eye coordination, and fine Motor Skills.

*Social Cognitive assessment* by the Edinburgh Social Cognition Test^57^, The Edinburgh Social Cognition Test (ESCoT) measures four social cognitive abilities: cognitive Theory of Mind; affective Theory of Mind; interpersonal understanding of social norms, and intrapersonal understanding of social norms. It consists of 11 dynamic, cartoon-style social interactions (each approximately 30 s long): 1 practice interaction, 5 interactions involving a social norm violation, and 5 portray everyday interactions that do not involve social norm violations. Each animation has a different context and specific questions relating to that context. The animations will run on most media players, but we recommend VLC due to ease of use. Each question is awarded a maximum of 3 points, resulting in a score of 12 points for each social interaction. The total maximum score for each subtest is 30 while the total score is out of 120.

*Laboratory assessment of metabolic markers* This was done to both groups (patients and control) by enzyme-linked immunosorbent assay (ELISA). We used the modified definition of the WHO criteria^58^, consisting of hyperinsulinemia (the upper fourth of the fasting insulin level among nondiabetic subjects) or hyperglycemia (fasting glucose ≥ 110 mg/dl) in addition to at least two of the following: waist girth ≥ 94 cm, dyslipidemia (triglycerides ≥ 150 mg/dl or HDL cholesterol ˂40 mg/dl), or BP ≥ 140/90 mmHg or taking BP medication. Waist circumference was measured by a tape at the central point between the lowest front rib and the highest front point of the pelvis on light clothing. The metabolic profile was determined using routine standardized laboratory methods. Lipid profile, blood glucose, and HbA1C samples were taken after 12 h of fasting at 9 a.m. Also, the body mass index was assessed by body weight in Kilograms divided by height on a meter square.

### Ethical consideration

A) Institutional Review Board approval of the local ethical committee of Hayat National Hospital, Riyadh, Kingdom of Saudi Arabia was taken to conduct this study and all the steps were in parallel with the Declaration of Helsinki (Reference No: HNH, 132-10-2020)
B) Informed consent was obtained from all participants after a full explanation of the aim of the study.
C) Patients confirmed the confidentiality of their data collected and that they were able to withhold from the study at any time without giving reasons.

### Statistical analysis

Data were fed to the computer and analyzed using SPSS^59,60^. Statistics for Windows, Version 22.0. Armonk, NY: IBM Corp. Qualitative data were described using numbers and percentages. Quantitative data were described using median (minimum and maximum) and mean, and standard deviation for parametric data after testing normality using the Kolmogrov-Smirnov test. The significance of the obtained results was judged at the (0.05) level.

### Data analysis

#### Qualitative data

Chi-Square and Monte Carlo tests for comparison of 2 or more groups.

#### Quantitative data between groups

##### Parametric tests

Student t-test was used to compare 2 independent groups.

### Spearman's correlation

Spearman's rank-order correlation is used to determine the strength and direction of a linear relationship between two non-normally distributed continuous variables and/or ordinal variables.

*Binary stepwise logistic regression* analysis was used for the prediction of independent variables of a binary outcome. Significant predictors in the Univariate analysis were entered into the regression model using the forward Wald method /Enter. Adjusted odds ratios and their 95% confidence interval were calculated.

*Linear regression analysis* was used for the prediction of independent variables of the continuous parametric outcome. Significant predictors in the correlation were entered into the regression model with the calculation of R2 the quantity effect of combined variables on the desired outcome and the prediction equation (Y = β + a*x).

### Informed consent

Informed consent was obtained from all participants after a full explanation of the aim of the study.

### Approval of the research protocol by the institutional reviewer bard

Institutional Review Board approval was taken to conduct this study and all the steps were in parallel with the Declaration of Helsinki (Reference No: HNH,132-10-2020).

## Results

The sociodemographic and clinical characteristics are illustrated in Table 1 in which the number of cases was 150 while the control group was 120, the mean age of the cases was (31.86 SD ± 9.06), 103 (68.7%) of the cases were males while 47 (31.3%) were females. the mean age of the control was (33.79 ± 8.77), 85 (70.8) of the control group were males while 35 (29.2) were females. There was a statistically significant difference between the studied groups regarding the employment status (p < 0.001), also the smoking status was of significant difference between the cases and control groups (p < 0.001). The family history of psychosis was in 73 cases (48.7%).

**Table 1:** Comparison of sociodemographic and clinical characteristics between studied groups.

	Control group n = 120	Cases group n = 150	Test of significance
Age/years	33.79 ± 8.77	31.86 ± 9.06	t = 1.77 p = 0.08
Sex			
Male	85(70.8)	103(68.7)	χ^2^ = 0.148
Female	35(29.2)	47(31.3)	p = 0.700
Marital status			
Single	53(44.2)	63(42)	χ^2MC^ = 2.13
Married	51(42.5)	59(39.3)	p = 0.544
Divorced	12(10)	24(16)	
Widowed	4(3.3)	4(2.7)	
Employment			
Employed	81(67.5)	65(43.3)	χ^2^ = 15.68
Unemployed	39(32.5)	85(56.7)	p < 0.001*
Residence			
Urban	85(70.8)	103(68.7)	χ^2^ = 0.148
Rural	35(29.2)	47(31.3)	p = 0.700
Education			
Elementary	49(40.8)	67(44.7)	χ^2^ = 1.87
University	58(48.3)	61(40.7)	p = 0.392
Higher education	13(10.8)	22(14.7)	
Smoking			
YES	36(30.0)	83(55.3)	χ^2^ = 1736
NO	84(70)	67(44.7)	p < 0.001*
Duration since onset			
0–3 months	Not applicable	97(64.7)	
3–6 months		53(35.3)	
Family history of psychosis			
Yes	Not applicable	73(48.7)	
No		77(51.3)	

t: Student t test χ^2^ = Chi-Square test, *MC* Monte Carlo test, *Statistically significant parameters described as mean ± SD, OR number and percentage.

The results of the PANSS test for the studied cases group were illustrated in the Table 2 in which the mean positive domain was 41.96 SD ± 3.28, the mean negative domain of PANSS was 43.73 ± 3.21, while the mean general domain was 97.16 ± 7.46 and the mean global score ± SD, it was 182.85 ± 9.16.

**Table 2:** PANSS score among studied cases.

PANSS	Cases group n = 150
Positive domain of PANSS	41.96 ± 3.28 (33–48)
Negative domains of PANSS	43.73 ± 3.21 (3.21–49)
General Domain of PANSS	97.16 ± 7.46 99(78–110
Global domain of PANSS	182.85 ± 9.16 (157–203)

*PANSS* positive and negative symptoms scale, parameters described as mean ± SD, median (min–max).

Physical findings and cardiometabolic parameters in the studied groups:

The physical findings and the cardiometabolic parameters between the studied groups were illuminated in a Table 3 in which all findings were of statistical significance (p˂0.001). BMI (t = 17.71. p < 0.001) and mean waist circumference (t = 16.70, p < 0.001) were of statistical significance. Also, both SBP (t = 20.88, p < 0.001) and DBP (t = 22.14, p < 0.001) were of statistically significant difference. Total cholesterol, TGS, LDL, and HDL were evident as shown in Table 3.

**Table 3:** Comparison of metabolic findings between studied groups.

	Control group n = 120	Cases group n = 150	Test of significance
BMI (kg/m^2^)	21.68 ± 2.32	29.55 ± 4.39	t = 17.71 p < 0.001*
Waist circumference/cm	88.40 ± 4.81	99.83 ± 6.14	t = 16.70 p < 0.001*
SBP (mm/HG)	125.57 ± 7.07	150.92 ± 11.70	t = 20.88 p < 0.001*
DBP (mm/HG)	81.60 ± 5.28	101.53 ± 8.65	t = 22.14 p < 0.001*
Total cholesterol	127.13 ± 11.88	234.72 ± 41.25	t = 27.66 p < 0.001*
LDL	64.58 ± 9.05	89.47 ± 14.50	t = 16.41 p < 0.001*
TGS	132.33 ± 19.03	260.66 ± 49.77	t = 26.72 p < 0.001*
HDL	93.71 ± 16.52	34.68 ± 11.02	t = 35.07 p < 0.001*
RBG	84.68 ± 11.28	152.34 ± 26.22	t = 26.38 p < 0.001*
HBA1c%	5.91 ± 0.95	9.13 ± 2.29	t = 14.40 p < 0.001*

t: Student t test *statistically significant, parameters described as mean ± SD.

*BMI* body mass index, *SBP* systolic blood pressure, *DBP* diastolic blood pressure, *TGS* triglycerides, *HDL* high density lipoprotein, *RBG* random blood glucose, *HBA1c* hemoglobin A1c.

The cases had higher levels of RBG (t = 26.38, p < 0.001) and HBA1C (t = 14.40 p < 0.001) than the control group.

Table 4 illustrated the cognitive and executive tests in which all its compared items were of statistical significance (p < 0.001), the flanker test in which the cases have more total errors than the control group (t = 33.6 p < 0.001).

**Table 4:** Comparison of cognitive and executive tests between studied groups.

	Control group n = 120	Cases group n = 150	Test of significance
1. Flanker			
Total errors	1.583 ± 0.87	5.36 ± 0.95	t = 33.6 p < 0.001*
Mean response time	635.95 ± 104.85	1108.28 ± 364.89	t = 13.73 p < 0.001*
Congruent time (ms)	600.91 ± 78.31	769.04 ± 126.49	t = 12.74 p < 0.001*
Incongruent time (ms)	617.89 ± 70.07	736.02 ± 118.49	t = 9.65 p < 0.001*
Conflict cost (incongruent mean-congruent mean)	16.82 ± 55.36	-33.87 ± 61.84	t = 7.01 p < 0.001*
2. Go/No Go test:			
Total correct responses	286.51 ± 13.36	101.09 ± 25.04	t = 73.19 p < 0.001*
Total errors responses	33.74 ± 13.43	218.91 ± 25.04	t = 73.01 p < 0.001*
Mean accuracy	0.895 ± 0.04	0.316 ± 0.078	t = 73.20 p < 0.001*
Mean error	0.114 ± 0.088	0.681 ± 0.085	t = 53.37 p < 0.001*
Mean response time in round 1	637.33 ± 87.98	1185.39 ± 378.30	t = 15.53 p < 0.001*
Mean response time in round 2	634.58 ± 89.51	1208.89 ± 364.72	t = 16.84 p < 0.001*
3. Simon test			
Correct answer	111.62 ± 11.30	56.22 ± 11.22	t = 40.18 p < 0.001*
Incorrect answer	28.30 ± 11.29	83.77 ± 11.19	t = 40.30 p < 0.001*
Reaction time	1014.67 ± 181.02	2111.13 ± 350.19	t = 31.13 p < 0.001*
4. Tower of London			
Total moves (ms)	113.53 ± 16.95	180.71 ± 14.78	t = 34.76 p < 0.001*
Total time (ms)	600.45 ± 89.73	973.20 ± 239.72	t = 16.15 p < 0.001*

t: Student t test, *statistically significant if p < 0.05, parameters described as mean ± SD.

Go/and No go test including all domains was significant between studied groups (p < 0.001), Fewer correct answers of the Simon test was in the cases group than in control one (t = 40.18 p < 0.001), and more incorrect answers (t = 40.30 p < 0.001). The patients had slow total moves (t = 34.76 p < 0.001) and total time(t = 16.15 p < 0.001) of Tower of London.

The domains of social cognition of the studied groups were described in Table 5.in which the cases group has the defective cognitive theory of mind (t = 56.91 p < 0.001), and impaired affective theory of mind (t = 65.48 p < 0.001).

**Table 5:** Comparison of mean social cognitive score between studied groups.

Social cognitive	Control group n = 120	Cases group n = 150	Test of significance
Cognitive theory of mind	27.52 ± 1.26	13.88 ± 2.37	t = 56.91 p < 0.001*
Affective theory of mind	28.49 ± 0.98	13.19 ± 2.40	t = 65.48 p < 0.001*
Interpersonal understanding	28.93 ± 0.99	13.83 ± 2.23	t = 68.81 p < 0.001*
Intrapersonal understanding	29.28 ± 0.82	13.83 ± 2.31	t = 69.71 p < 0.001*
Total score	114.22 ± 2.09	54.73 ± 4.44	t = 135.26 p < 0.001*

t: Student t test, *statistically significant if p < 0.05, parameters described as mean ± SD.

The cases group had more impaired interpersonal (t = 68.81 p < 0.001) than the control group. The intrapersonal understanding of social norms was significant in the studied groups (t = 69.71 p < 0.001).

Table 6 showed that the mean affective theory of mind is negatively correlated with the conflict cost of the Flanker test (r = − 0.185* p value = 0.023).

**Table 6:** Correlation between flanker test and Social cognitive score.

Flanker test							
			Total errors	Mean response time	Congruent time	Incongruent time	CONFLICT COST
Social cognitive score	Cognitive theory of mind	r	0.021	0.067	0.050	0.078	0.048
		p value)	0.801	0.417	0.545	0.344	0.559
	Affective theory of mind	r	0.031	0.043	0.105	0.030	− 0.185*
		p value)	0.704	0.604	0.199	0.716	0.023
	Interpersonal understanding	r	− 0.035	− 0.044	0.088	0.104	0.035
		p value)	0.673	0.596	0.282	0.206	0.675
	Intrapersonal understanding	r	0.000	− 0.020	− 0.016	− 0.005	0.062
		p value)	0.995	0.807	0.843	0.951	0.451
	Total score	r	0.020	0.055	0.132	0.106	− 0.052
		p value)	0.804	0.504	0.106	0.195	0.530

r: Speraman correlation coefficient.

*, **Statistically significant.

Table 7 illustrated that the elevated levels of total cholesterol (r = − 0.241**, p value = 0.003), and triglycerides (r = − 0.207*, p value = 0.011) had a negative correlation to the score of the interpersonal domain of social cognition Also, the elevated total cholesterol is positively correlated to the total score of social cognition (r = 0.202*, p value = 0.013).

**Table 7:** Correlation between metabolic profile and social cognitive functions in the cases group:

		Total cholesterol	LDL	TGS	HDL	RBG	HBA1c
Cognitive theory of mind	r	0.049	− 0.051	0.061	0.051	0.101	− 0.145
	p	0.554	0.536	0.455	0.538	0.218	0.077
Affective theory of mind	r	0.044	0.075	− 0.005	0.062	0.112	0.091
	p	0.594	0.363	0.948	0.448	0.172	0.266
Interpersonal understanding	r	− 0.241**	0.092	− 0.207*	0.030	0.075	0.152
	p	0.003	0.265	0.011	0.714	0.363	0.063
Intrapersonal understanding	r	0.059	0.085	0.074	− 0.105	− 0.100	0.006
	p	0.472	0.299	0.371	0.202	0.226	0.946
Total score	r	0.202*	0.104	-0.036	0.021	0.100	0.052
	p	0.013	0.206	0.663	0.795	0.221	0.531

*LDL* low density lipoprotein = Triglycerides, *HDL* high density lipoprotein, *RBG* random blood glucose, *HBA1c* hemoglobin A1C.

The binary logistic regression was illustrated in the Table 8 in which the prediction of the risk factors in the studied cases of 1st episode psychosis in which unemployment (β = 1.049, p-value < 0.001*, odds ratio (95% CI) 2.85 (1.69–4.80), smoking (β = 1.11, p-value < 0.001*, odds ratio (95% CI) 3.03 (1.79–5.12) were associated with the outcome. BMI (kg/m^2^) (β = 0.440, p-value 0.005*odds ratio (95% CI) 1.55 (1.14–2.11), Waist circumference/cm (β = 0.352, P-value 0.006*odds ratio (95% CI) 1.42 (1.10–1.83) which were 2 parameters of metabolic assessment represent predictive factors of social cognition in those patients. In the same vein, Diastolic Blood Pressure (mm/HG) (β = 0.301, p-value 0.015* odds ratio (95% CI) 1.35 (1.06–1.72), total cholesterol (β = 0.219, p-value 0.003* odds ratio (95% CI) 1.24 (1.08–1.44), Low-Density Lipoprotein (β = 0.189, p-value 0.01* odds ratio (95% CI) 1.20 (1.04–1.39) were predictors of social cognition in 1st episode patients with psychosis. Also, the reaction times of the Simon test (β = 0.002, p-value 0.998, odds ratio (95% CI) 1.002), the total score of Social cognition (β = − 0.722, p-value 1.0, odds ratio (95% CI) 0.486), the total reaction time of Tower of London (β = 0.004, p-value 1.0, odd ratio (95% CI) 1.004) and total errors of Flanker test (β = 0.209, p-value 1.0, odds ratio (95% CI) 1.232) were predictive factors of social cognition in the cases group.

**Table 8:** Binary logistic regression for prediction of the studied cases.

	β	p value	odds ratio (95%CI)
Employment			
Un employed	1.049	< 0.001*	2.85 (1.69–4.80)
Smoking	1.11	< 0.001*	3.03 (1.79–5.12)
BMI (kg/m^2^)	0.440	0.005*	1.55 (1.14–2.11)
Waist circumference/cm	0.352	0.006*	1.42 (1.10–1.83)
SBP (mm/HG)	0.140	0.127	1.15 (0.961–1.38)
DBP (mm/HG)	0.301	0.015*	1.35 (1.06–1.72)
TOTAL CHOLESTEROL	0.219	0.003*	1.24 (1.08–1.44)
LDL	0.189	0.01*	1.20 (1.04–1.39)
Simon test: reaction times	0.002	0.998	1.002 (undefined)
Social cognition: total score	-0.722	1.0	0.486 (undefined)
Tower of London: total reaction time	0.004	1.0	1.004 (undefined)
Flanker test: total errors)	0.209	1.0	1.232 (undefined)
Overall % predicted = 55.6%			

*BMI* body mass index, *SBP* systolic blood pressure, *DBP* diastolic blood pressure, *LDL* low density lipoprotein, *CI* confidence interval.

## Discussion

This study aimed to evaluate the metabolic parameters in patients with 1st episode psychotic disorders and the relationship between these parameters and cognitive, executive functions, and social cognition.

Laboratory evaluation was done for assessment of the lipid profile, glycemic status, central obesity, and blood pressure among the patients with psychotic features and the healthy control group.

This study revealed that smoking was common in the patients with 1st episode psychosis which was in parallel with^61,62^ who noted the smoking prevalence in their studies samples was (55%) and, in another study it was smoking was 47% observed in FEP^63^, but is considerably lower than the more recent, Gaughran et al.^64^ and Manzella et al.^65^ studies which revealed much higher rates of cigarette smoking (76.8% and 78% respectively) in FEP patients. These higher rates of smoking in FEP were compared to rates of 19%– 21% in the age-matched general population.

The patients with 1st episode psychosis had a higher level of BMI/central obesity, abdominal adiposity, elevated blood pressure, and higher lipid profile including cholesterol, TGS, LDL, and lower HDL which reflected the cardiometabolic parameters in 1st episode psychosis^66,67^.

Also, of the patients with FEP, 58% of them had dyslipidemia, 40% had hypertriglyceridemia, and 30% of participants met the criteria of metabolic syndrome, also high triglycerides, typically elevated in FEP, are a precursor for T2DM^67^. Hypertension and blood glucose harm the immediate and recognition memory domains of cognitive functions^68^. Waist circumference as a parameter of assessment of obesity per se was negatively correlated with impairment in different cognitive domains including processing speed, attention, working memory, and reasoning which was noted in a previous similar study^69^.

The patients of 1st episode psychotic disorders had worse scores on multiple cognitive tests which were in line with previous studies that noted cognitive impairments in the drug naïve patients with psychosis^70–72^. In the same vein, the results of the study are in parallel with the studies^73^ that found a significant negative correlation between cognitive functions and triglycerides which was associated with poorer working memory in FEP.

Also, the cognitive performance of a patient with first-episode psychotic features particularly cognitive flexibility, attention, memory, and current IQ was negatively associated with the domains of physical health, including body mass index (BMI) and waist circumference^74,75^.

On the other hand, the metabolic changes were more commonly reported in chronic schizophrenic patients than in 1st episode psychotic ones which were attributed to the metabolic derangements caused by the effects of antipsychotic medications on the metabolic profiles and glycemic status^76^.

As other authors have illustrated, cognitive functions may not be associated with cardiometabolic parameters, and the impairment of cognitive functions occurred only with severe disturbance of glucose metabolism and predisposition to diabetes mellitus^77^. On the contrary, other studies^78^ revealed no relation between cognitive performance including working memory, attention, strategic planning, and the different metabolic parameters especially the random blood glucose and HBA1C which represented crucial determinants of Diabetes Mellitus. Previous studies concluded that FEP patients showed impairments in different domains of social cognitive functions including TOM, and interpersonal and intrapersonal understanding^79–81^.

Also, the results of^82,83^ confirmed our results that lower scores of domains of social cognition were positively correlated to deteriorated performance on cognitive and neuropsychological tests which reflected on attention, memory, planning, decision-making, and mental flexibility.

The scores of social cognitions in patients with psychosis including the theory of mind, interpersonal domain, and the total score were inversely correlated to HBA1C and random blood glucose^84^. The level of triglycerides, cholesterol, BMI, and waist circumference as metabolic parameters were negatively correlated to the different domains of social cognition including TOM which were in line with a similar study but it was conducted on the schizophrenia spectrum disorders^85^.

On the other point of view, other studies^86–88^ found no statistically significant differences between the mean scores of different subdomains of social cognitive battery in FEP patients and healthy controls. On the contrary^89^, found in their studies that there was no correlation between the different domains of the metabolic profile including waist circumference, lipid profile, blood pressure, and glycemic status with the subdomains of social cognition.

A multiple linear regression analysis was conducted and showed that cognitive tests and social cognition were correlated with BMI, waist circumference total cholesterol, DBP, and TG. These findings were in line with previous studies that random blood glucose and blood pressure levels were significantly associated with cognitive and social cognition disturbance in processing speed, verbal learning, visual learning, and executive ability in 1st episode patients with psychosis^90–92^.

## Conclusions

In conclusion, the metabolic profile in 1st episode patients with psychosis showed disturbance of physical health, lipid profile, blood pressure, and glycemic status. Also, the cardiometabolic parameters have an association with cognitive and social cognition.

### Study limitations

The study needs a greater number of cases for generalization of the results, also the assessment was done once at the initial phase of the disorder, more frequent evaluation will be needed after initiation of the medication and implementation of a healthy lifestyle to assess their effects on cognitive functions and social cognition.

## Data Availability

The data are available upon request from the corresponding author.

## Abbreviations

APA: American Psychiatric Association
BMI: Body mass index
BPRS: Brief Psychiatric Rating Scale
CR: Cognitive reserve
DBP: Diastolic blood pressure
ELIZA: Enzyme-linked immunosorbent assay
ESCoT: Edinburgh Social Cognition Test
FEP: First episode psychosis
HDL: High-density lipoprotein
LDL: Low-density lipoprotein
MAs: Metabolic abnormalities
PANSS: Positive and negative symptoms scale
PRS: Psychopathology Rating Schedule
RBG: Random blood glucose
SBP: Systolic blood pressure
TGs: Triglycerides
TOL: Tower of London
TOM: Theory of mind
